# Detecting Foci of Malaria Transmission with School Surveys: A Pilot Study in the Gambia

**DOI:** 10.1371/journal.pone.0067108

**Published:** 2013-06-27

**Authors:** Ebako N. Takem, Muna Affara, Alfred Amambua-Ngwa, Joseph Okebe, Serign J. Ceesay, Musa Jawara, Eniyou Oriero, Davis Nwakanma, Margaret Pinder, Caitlin Clifford, Makie Taal, Momodou Sowe, Penda Suso, Alphonse Mendy, Amicoleh Mbaye, Chris Drakeley, Umberto D'Alessandro

**Affiliations:** 1 Medical Research Council Unit, Fajara, The Gambia; 2 University of Manchester, Manchester, United Kingdom; 3 National Public Health Laboratory, Kotu, The Gambia; 4 Ministry of Basic and Secondary Education, Banjul, The Gambia; 5 London School of Hygiene and Tropical Medicine, London, United Kingdom; 6 Institute of Tropical Medicine, Antwerp, Belgium; Université Pierre et Marie Curie, France

## Abstract

**Background:**

In areas of declining malaria transmission such as in The Gambia, the identification of malaria infected individuals becomes increasingly harder. School surveys may be used to identify foci of malaria transmission in the community.

**Methods:**

The survey was carried out in May–June 2011, before the beginning of the malaria transmission season. Thirty two schools in the Upper River Region of The Gambia were selected with probability proportional to size; in each school approximately 100 children were randomly chosen for inclusion in the study. Each child had a finger prick blood sample collected for the determination of antimalarial antibodies by ELISA, malaria infection by microscopy and PCR, and for haemoglobin measurement. In addition, a simple questionnaire on socio-demographic variables and the use of insecticide-treated bed nets was completed. The cut-off for positivity for antimalarial antibodies was obtained using finite mixture models. The clustered nature of the data was taken into account in the analyses.

**Results:**

A total of 3,277 children were included in the survey. The mean age was 10 years (SD = 2.7) [range 4–21], with males and females evenly distributed. The prevalence of malaria infection as determined by PCR was 13.6% (426/3124) [95% CI = 12.2–16.3] with marked variation between schools (range 3–25%, p<0.001), while the seroprevalence was 7.8% (234/2994) [95%CI = 6.4–9.8] for MSP1_19_, 11.6% (364/2997) [95%CI = 9.4–14.5] for MSP2, and 20.0% (593/2973) [95% CI = 16.5–23.2) for AMA1. The prevalence of all the three antimalarial antibodies positive was 2.7% (79/2920).

**Conclusions:**

This survey shows that malaria prevalence and seroprevalence before the transmission season were highly heterogeneous.

## Background

Malaria is currently endemic in 104 countries and territories around the world, and is a major health problem in many countries in sub-Saharan Africa [1]. The importance of monitoring the burden of malaria and the effectiveness of control efforts cannot be overemphasized. Monitoring can be done using routine surveillance data from health facilities and household surveys, the latter providing information on indicators such as coverage of vector control methods (long lasting insecticide treated nets and indoor residual spraying), malaria transmission or malaria mortality [2]. Household surveys include demographic and health surveys, multiple indicator cluster surveys, and malaria indicator surveys, all requiring important human and financial resources. Therefore, alternative and simple methods, to be carried out at more regular intervals for rapidly monitoring malaria trends, would be extremely helpful.

School surveys are attractive, as both their location and target population are easily accessible, with often high compliance [3]. Their cost is usually much lower than that of more classical approaches while the age groups surveyed overlaps with the one classically used to define endemicity, i.e. the 2–9 years old group [4], [5]. In addition, in areas where transmission has substantially decreased, the malaria risk is higher in 5–14 years old children than in the <5 years old [6], [7]. In sub-Saharan African countries, malaria school surveys have been carried out for different purposes [8]–[17]. In Uganda, they were used to determine bed net coverage in the community [12] while in Kenya, besides providing data on vector control interventions coverage and acceptability of a malaria control strategy, school surveys were used to estimate the malaria burden, its relationship to anaemia and the incidence of clinical malaria [8], [11], [15], [18]. In other countries, e.g. The Democratic Republic of Congo [9] and Ethiopia [16], school surveys provided information on the malaria geographical heterogeneity and helped identifying clusters of transmission. Their representativeness, as compared to the whole community, will obviously depend on the degree of school attendance.

In the past few years, a reduction of the burden of malaria has been described in some African settings [19]–[22]. In The Gambia, a substantial decline in the burden of malaria was described from 1999 to 2009 [23], [24]. As a result, malaria elimination is being considered as a possibility.

In areas of declining transmission such as The Gambia, where the identification of malaria infected individuals becomes increasingly harder, school surveys may represent an economic and easy method to identify communities where malaria transmission remains substantial. We hypothesise that school surveys can be used to detect foci of malaria transmission in the community.

## Methods

### Study Population and Design

The study was conducted in the Upper River Region (URR) in The Gambia, West Africa, where malaria transmission is seasonal and occurs mainly during the “wet” months (July-December). Most of the malaria burden is due to *P.falciparum* infections. In 2007–2008, the incidence of malaria in children in this region was estimated at 0.79 per child year [25]. Since 2008, the first line antimalarial treatment is the combination artemether-lumefantrine (AL). *Anopheles gambiae* is the main vector, with increased vector density between August-September and an estimated annual entomological inoculate rate of 2.27 [26], [27].

The survey was carried out before the beginning of the transmission season, in May/June 2011, and lasted for two weeks. Children were selected using a two-stage sampling process; first, primary schools in URR were selected with probability proportional to size (according to the size of the school), and then secondly, in each school, about 100 children were randomly chosen. The sampling process was done using the random generator on the Microsoft Excel package. All children attending the selected schools were eligible, regardless of the age, though it was expected that the majority of them would fall in the 5–14 years age group. Children involved in another research project at the time of the survey were excluded.

### Ethics Statement

An information sheet, explaining the objectives of the survey and the study procedures, was distributed, prior to the survey, to the children’s parents who were asked to provide a written informed consent on the day of the survey.

The study proposal was reviewed and approved by the Scientific Coordinating Committee of the Medical Research Council Unit, The Gambia, and subsequently ethical approval was granted by The Joint Gambia Government/Medical Research Council Ethics committee.

### Data Collection

Data on demographic characteristics and on reported use of bed nets, including long-lasting Insecticidal nets (LLIN), were collected using a structured questionnaire on the case report form (CRF). This was done by interviewing the parent or carer of each child. Weight and height were measured, the former using a bathroom scale and the latter a stadiometer (Leicester portable Height Measure). For each child, a finger prick blood sample was collected for thick smear, haemoglobin (Hb) measurement, and two blood spots on filter paper (Whatman 3 MM) for later serology and molecular analysis. Filter paper blood spots were air dried and stored in plastic bags containing a desiccant. In case of fever (body temperature ≥37.5°C), a rapid diagnostic test (ICT malaria cassettes, ICT Diagnostics, South Africa) for *P.falciparum* antigens was performed and, if positive, the child was treated with AL.

### Laboratory Methods

Antimalarial antibodies against merozoite surface protein-1_19_ (MSP1_19_), MSP2, and apical membrane antigen-1 (AMA1), were detected by indirect ELISA on eluted filter paper samples. Discs (3 mm), two for each sample, were punched and placed at the bottom of 96 well plates. Serum was eluted from dried blood spots (DBS) following overnight (18 h) incubation at room temperature in 150 µL of reconstitution buffer (150 µl Phosphate Buffer Saline-PBS/0.05% (v/v) Tween20/0.05% (w/v) NaN3) with gentle mixing (rotary shaker at 200 revs per min). The reconstituted solution was equivalent to a 1/100 dilution of whole blood, corresponding to 1∶200 dilution of plasma or serum antibodies, if the blood were at 50% haematocrit. For ELISA, antigens were coated on Immunolon HBX plate at a concentration of 5 µg/ml and each reconstituted sample was further diluted fivefold (final serum dilution of 1∶1000) and analysed in duplicate wells. Standard ELISA procedures for blocking unbound sites, washing of unbound antigens and antibodies, and incubation were followed as per described DBS protocols [28], [29]. Each ELISA plate included a serum standard constituting a fivefold serial dilution of malaria positive control serum from Brefet, The Gambia [30]. Negative controls included un-exposed European pooled serum and plate blanks (no serum). Plates were developed with HRP conjugated anti-human antibodies and O-phenylenediamine (OPD) substrate following manufacturer’s instructions (Sigma). Optical densities were read on GeminiXPS microplate reader and converted to antibody units using 5-parameter curves from reference standards generated with the MasterPlex reader fit software.

Molecular diagnosis for malaria parasites and speciation of Plasmodium spp. employed DNA extracted from DBS on the QIA X-tractor robot following the manufacturer’s protocol (Qiagen). Briefly, 3 mm discs punched from each sample were placed at the bottom of 96 well plates and treated with tissue digest buffer at 60°C for 1 hour. DBS digest eluates were then applied on capture plates, washed and DNA eluted with 100 µL of elution buffer. Diagnostic PCR followed amplification of the multi copy Plasmodium ribosomal RNA gene sequences using genus and species specific primers, as previously described [31], [32]. Amplified fragments were separated and analysed on the Qiaxcel automated capillary electrophoresis system using either the FastAnalysis or Screening catridges with 25–3000 bp or 15–1000 bp alignment markers respectively. PCR band sizes, to determine species of infection, were determined against a 25–800 bp ladder analysed on each 96 well plate. Unless stated otherwise, the reported parasite prevalence is that determined by PCR.

Blood slides corresponding to all PCR positive samples were read by microscopy in order to determine the parasite density. In addition, 30 randomly chosen PCR negative samples were included to assess the agreement between microscopy and PCR. Thick films for microscopy were prepared by placing two drops of peripheral blood on the centre of the slide. Films were air dried horizontally, and left to dry overnight and then stained with Giemsa(1∶10 dilution for 10 minutes). Two hundred high power fields (HPF) were read before declaring the slide negative. If positive, the number of parasites against HPF was counted until 200 HPF were screened. The parasite density per µl was determined assuming 500 HPF per µl [33]. Each slide was read independently by two trained microscopists and the average count taken if the difference was less than 10-fold. In case the difference was more than 10 fold, a third reading by a senior microscopist was considered the final result.

Haemoglobin (Hb) was measured with the Hemocue 301 machine (Angelholm, Sweden) following the manufacturer’s instructions.

### Sample Size

The lowest seroprevalence of antimalarial antibodies in a previous survey carried out in the same area in January/February 2008 was around 20% [7]. One thousand five hundred and thirty seven participants (rounded to 1,600) would be sufficient to estimate such seroprevalence with a precision of 2% and with 95% confidence limits. Since the sampling was done within clusters (schools) and assuming a design effect of about 2, the final sample size was estimated at 3,200 children, with clusters of 100 children each, i.e. 32 schools.

### Data Entry and Statistical Analyses

Data from the CRFs was double-entered into a database (OpenClinica, Akaza Research, USA). After cleaning, the data was analysed with STATA version 12 (College Station, Texas). Means and standard deviation were estimated for quantitative variables. The variable’s distribution was examined using histograms, and in case this was skewed, the variable was log transformed for the estimation of the geometric mean. Kernel distribution curves were also used to study the distribution of quantitative variables, particularly the antibody titres. In case of categorical variables, proportions in tables were used to describe the variables. Anthropometric measurements for height and weight were used to estimate the weight-for-age (wfaz), height-for-age (hfaz) and Body Mass Index-BMI for age (bfaz) z scores, by comparing the survey data to WHO growth reference data for school-age children and adolescents [34].

The cut-off for positivity for antimalarial antibodies was obtained by modelling their normalized value (log transformed), using finite mixture models - Gaussian distribution with two components [29]. The estimated mean of the narrower distribution was used as the mean for negative samples. The cut-off for seropositivity was obtained by taking the difference between the antibody titres and the mean of the narrower distribution plus 3 standard deviations [29]. The proportion of seropositives for a specific malaria antigen (seroprevalence) was then estimated.

Seroprevalence rates were analysed according the following age groups: <8 years, 8–9, 10–11, ≥12 years so there would be a similar number of children in each group. The seroprevalence in each age group was obtained using proportions in contingency tables. Regression models (logistic) were also used with age as a continuous variable. A line of best fit, using the quadratic estimation, was used to obtain the predicted probability of being seropositive for a particular parasite antigen. A similar model was applied to obtain the age-specific PCR parasite prevalence.

The distribution of parasite, antimalarial antibodies (seroprevalence) and anaemia prevalence were estimated across schools, taking into account the clustered nature of the data. The data was set for survey design taking into account the two stage sampling with the number of schools and the total number of children in the sampling frame. The participants were arranged according to the geographical coordinates of the schools in order to determine spatial heterogeneity. The designed-based F test was used to determine if the difference across schools was significant (individual analyses).

The correlation between parasite prevalence and seroprevalence and the correlation between parasite prevalence across schools was examined by scatter plots. The correlation coefficient was estimated with the Spearman non parametric test, and a linear regression line of best fit was used to estimate the relationship between the two variables.

## Results

Among the 77 primary schools in URR attended by 16,554 children, 32 were selected and from these 3,277 children were enrolled into the survey. The mean age was 10 (SD = 2.7) years [range 4–21], with males and females evenly distributed, and a large majority of the children (72.5%, 2363/3261) used a bed net (Table 1). At the time of the survey, 108 (3.3%) children had fever, though a greater proportion (17.1%, 560/3274) claimed to have had fever in the past 48 hours. The prevalence of anaemia (Hb<11g/dl) was 10.5% (342/3261) and that of severe anaemia (Hb<7g/dl) 0.4% (12/3261). All measured nutritional indices were negative (Table 1), and a substantial proportion of children had z- scores <-2 SD (wfaz: 17.0%, hfaz: 11.7%, bfaz: 31.1%).

**Table 1:** Baseline characteristic and prevalence of malaria and anaemia.

Variables	%
Mean age in years (SD)	10.0 (2.73)
Male/Female	51.7/48.3 (1637/1532)
Bed net use	72.5 (2363/3261)
Mean weight in Kg (SD)	27.7 (8.2)
Mean height in cm (SD)	135.4 (13.0)
Weight for age z score-wfaz‡ (SD) N = 1,834	−0.79 (1.3)
Height for age z score-hfaz (SD) N = 3,034	−0.38 (1.5)
Mean BMI for age z score –bfaz (SD) N = 2,986	−1.4 (1.3)
History of fever in the past 48 h	17.1 (560/3274)
Fever (BT≥37.5°C)	3.3 (108/3269)
Mean Haemoglobin (SD)	12.4 (1.33)
Anaemia (Hb<11 g/dl)	10.5 (342/3261)
MSP1_19_ seroprevalence	7.8 (234/2994)
MSP2 seroprevalence	11.6 (346/2977)
AMA1 seroprevalence	19.9 (593/2973)
Plasmodium prevalence by PCR	13.6 (426/3124)
falciparum prevalence by PCR	8.9 (278/3124)
GM* Asexual parasite density per µl (SD)	18.25(7.28)**
GM* gametocyte density per µl(SD)	3.49(1.57)***

‡ wfaz reference values available only from 5–10 years and comparison was done for this age group. hfaz and bfaz estimated for 5–19 years.

* GM = Geometric mean.

** n = 340.

*** n = 20.

PCR analysis was carried out on 3,124 filter paper blood samples; 426 (13.6%) [95% CI = 12.2–16.3] were found to be positive for malaria infection, though only 278 (8.9%) were confirmed to be *Plasmodium falciparum* while for the remaining samples no other Plasmodium species (vivax, ovale, malariae) was found. Given the low parasite density in the survey samples, this discrepancy could have resulted from the greater sensitivity of the genus-specific PCR. Prevalence of infection varied with age, with the lowest values found in 8–9 years old children (11.8%, 98/828), and the highest in the 10–11 year old (15.3%, 122/798). Malaria prevalence tended to be higher in febrile (16.7%, 17/102) than (13.5%, 408/3014) afebrile children but the difference was not statistically significant (p = 0.44). No infection was detected by microscopy in 15.3% (65/424) of PCR positive samples, while in 2 out of 29 PCR negative samples (for one sample the blood slide was not found), microscopy identified a malaria infection. The mean parasite density in these 2 discrepant samples was extremely low (32 parasites per µl). Overall, there was 84.7% concordance between PCR and microscopy in the subset of samples screened by both methods.

**Figure 1:**
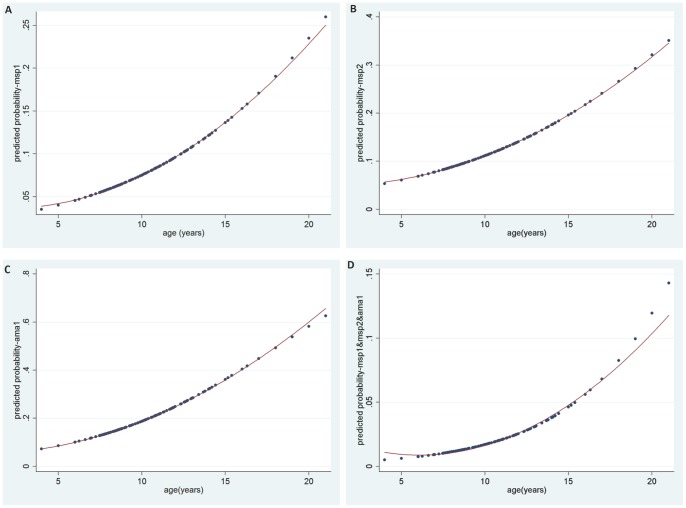
Age-specific seroprevalence. **A.** MSP1_19_. **B.** MSP2. **C.** AMA1. **D.** MSP1_19_, MSP2 and AMA1(combined).

**Figure 2:**
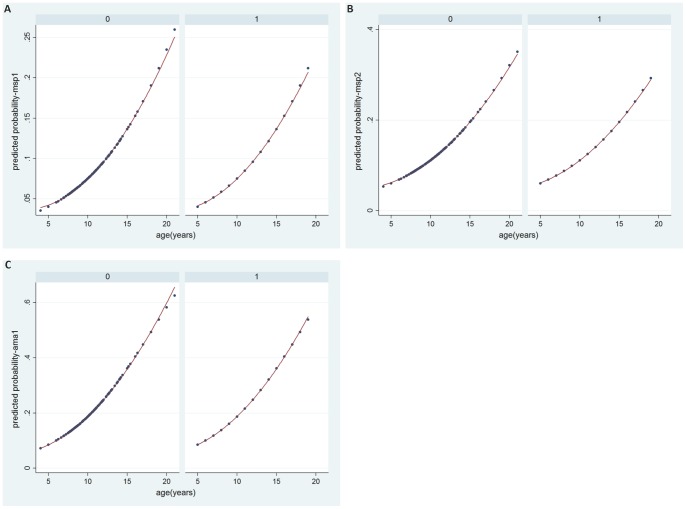
Age specific seroprevalence according to parasite prevalence (low versus high). **A.** MSP1_19_. **B.** MSP2. **C.** AMA1. 0 = Sites with plasmodium prevalence ≤20% (low). 1 = Sites with plasmodium prevalence >20% (high).

The prevalence of antimalarial antibodies (seroprevalence) ranged from 7.8% (234/2994) [95%CI = 6.4–9.8] for MSP1_19_, to 11.6% (364/2997) [95%CI = 9.4–14.5] for MSP2 and 20% (593/2973) [95% CI = 16.5–23.2) for AMA1 (Table 1). The proportion of children with at least one of the three antimalarial antibodies tested was 29% (826/2852) [95% CI = 25.1–33]. Most PCR negative samples were also negative for antimalarial antibodies (MSP1_19_: 93.5%, MSP2: 90.6%, AMA1: 82.2%). The proportion of children with all the three antimalarial antibodies positive was 2.7% (79/2920). Among the children with malaria infection detected by PCR, 16.4% were positive for MSP1_19_, 26.1% for MSP2 and 34.3% for AMA1. As expected, age-specific seroprevalence increased significantly with age for MSP1_19_ (from 4% in <8 years old to 12% in ≥12 years) (p<0.001), MSP2 (from 6% in <8 years old to 17% in ≥12 years)(p<0.001) and AMA1 (from 11% in <8 years old to 31% in ≥12 years)(p<0.001) - graphs not shown. After a regression model with age as a continuous variable, the quadratic fit appeared to be the most plausible with the increase in seroprevalence more pronounced in the older than in the younger participants (Figure 1A–C). A similar trend with age was observed for all the three antibodies combined (Figure 1D). The age-specific seroprevalence for each antigen also increased with age in high and low parasite prevalence sites (Figure 2A–C).

**Figure 3:**
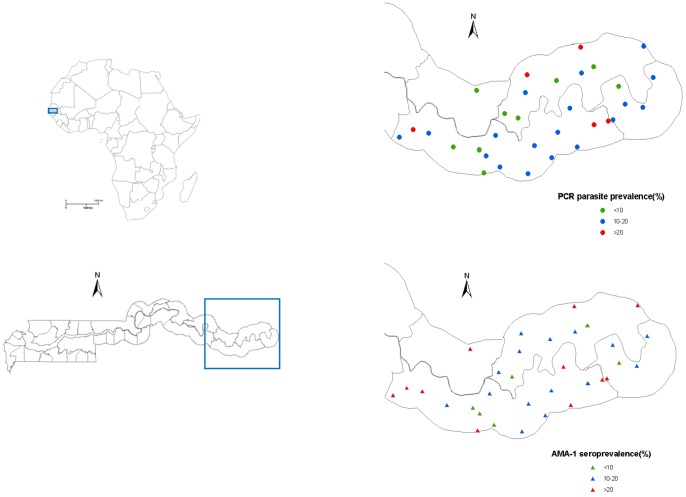
Parasite prevalence and seroprevalence by school.

Malaria prevalence varied substantially (range 4–26%) between schools (p<0.001), and was spatially heterogeneous, with low (<10%) and high (>20%) prevalence schools geographically contiguous (Figure 3). Similarly, seroprevalence varied between schools and ranged between 0% and 21% for MSP1_19_, 1% to 50% for MSP2 and 4% to 76% for AMA1. Differences between schools were statistically significant for MSP2 and AMA1 (p<0.001) but not for MSP1_19_ (p = 0.12), with the highest variability with AMA1. Though seroprevalence against the individuals antigens was correlated, this was stronger between MSP2 and AMA1 (r = 0.74) (p<0.001) than between MSP1_19_ and the other two antigens (r = 0.3).

Parasite prevalence and seroprevalence for each of the antigens were weakly correlated (r = 0.4, p = 0.02–0.03). Similarly, parasite prevalence and seroprevalence for at least one antigen were weakly correlated (r = 0.37, p = 0.04).

Malaria prevalence tended to be lower in children using a bed net (12.8%, 290/2260) than in the others (15.8%, 134/849) (p = 0.10) while the risk of anaemia (10.1%, 238/2352 versus 11.8%, 103/892) (p = 0.17) and severe anaemia (0.45%, 10/2352 versus 0.25%, 2/892) (p = 0.46) was similar between these two groups. The MSP1_19_, MSP2 or AMA1 seroprevalence did not differ according to bed net use (data not shown).

There was no association between the anthropometric indices (wfaz, hfaz,bfaz) and the risk of malaria infection (data not shown). However, anaemia was significantly associated with hfaz; its prevalence was significantly higher in children with low (13.2%, 131/1010) than in those with medium (9.2%, 92/1006) and high (8.4%, 85/1008) hfaz (p = 0.004).

## Discussion

The prevalence of malaria infection in school children in URR was higher than expected as the survey was carried out before the beginning of the malaria transmission season. The large majority of children were asymptomatic and carried infections of low parasite density. Since in The Gambia there is little or no malaria transmission during the dry season, between January and June, the infections detected in this survey were probably acquired during the previous transmission season and then carried for several months. These infections, mostly asymptomatic and therefore not treated, are probably responsible for restarting the transmission at the beginning of the rains, in June-July, when the vector density increases. Although higher than expected, the parasite prevalence in this study is still consistent with the recently reported decline in malaria transmission in The Gambia [23], [24]. Indeed, earlier studies (between 1986 and1993) in the same region found that the malaria prevalence in children <5 years old was 46–71.2% during the transmission season [35]–[37], and 45% in the 1996 dry season [38], while it was 0.5–1.3% [25], [39] in the 2007–2008 transmission seasons and 0.6–4.1%(≤15 years) in the 2009 dry season [6], indicating a major decrease in the malaria burden. The lower sensitivity of species specific PCR primers [31] at low parasite densities, as compared to genus specific PCR primers [40], may explain the discrepancy between the results of these two tests, i.e. positive Plasmodium but negative falciparum PCR. This is plausible as the falciparum negative samples but positive for Plasmodium were further analysed and none of the samples was positive for non-falciparum species.

Anti-MSP1_19_, MSP2 and AMA1 antibodies have been associated with protection against clinical malaria [38], [41]–[43] and are also an indication of recent exposure to malaria infection. The link between prevalence of infection and antimalarial antibodies has already been reported [38], [42]–[44]; in this survey the two variables were not strongly correlated, probably because the survey was carried out at the end of the dry season, after several months of virtually no transmission. Antibodies reflected cumulative exposure during the previous transmission seasons while current infections represented just a fraction of previously acquired ones, those that were able to remain asymptomatic for several months. Therefore, seroprevalence may help in identifying foci of transmission, assuming that these are relatively stable from year to year [45].

Prevalence of malaria infection and of antimalarial antibodies was substantially heterogeneous, showing that this is not a feature limited to the later phases of malaria elimination [46]. Indeed, heterogeneity of transmission can occur also in high transmission areas though this may be less obvious as most people would be infected [47]. The reasons for heterogeneity have not been completely elucidated but probably include factors related to the vector, the human host and the interaction of these two with the environment. Where malaria prevalence has decreased to relatively low levels, targeting hotspots with interventions such as LLIN could lead to malaria elimination while untargeted interventions employing the same resources would result in more modest reductions [47]. Therefore, defining simple methods for the detection of malaria transmission hotspots would make their targeting easier; both parasite and antibodies prevalences seem to be the most robust indicators for the detection of hotspots [47] and carrying school surveys may be an easy and attractive approach for their detection. Nevertheless, it needs to be further validated for this purpose as schools may be attended by children originating from different villages and thus may not have the required resolution to identify specific hotspots with precision.

Although the anthropometric indices wfaz, hfaz and bfaz, are related, each indicates some specific underlying processes or outcomes. In general, a low wfaz, commonly termed underweight, indicating a child is not gaining sufficient weight relative to the age, could be explained by wasting/stunting [48]. A low hfaz score or stunting indicates the child is not gaining sufficient height relative to age, and implies long term malnutrition or poor health. The bfaz is another weight-based score, and takes into account the weight and the height for a particular age. Generally, low anthropometric scores may be a consequence of low intake of nutrients through diet, excessive energy expenditure such as during illness, or insufficient absorption. The low anthropometric scores obtained in this survey are within the limits of values found in developing countries, including The Gambia [48]–[50]. There was no evidence that the anthropometric scores were associated to the risk of malaria infection. This might be due to the cross-sectional nature of the study since data on malaria infection and anthropometric parameters were collected at the same time. The relationship between malnutrition or undernutrition and the risk of malaria is controversial. Some previous studies have shown an association [51], [52] while most of the others did not [53]–[56].

### Conclusions

In conclusion, the prevalence of asymptomatic malaria infection in school children at the end of the dry season was heterogeneous, with some schools having a relatively high prevalence. Such infections have probably been acquired during the previous transmission season and carried for several months. In areas where transmission has decreased substantially over a relatively short period, such as The Gambia, these asymptomatic and low density infections will restart the transmission at the time the vector density increases, after the onset of the rains. Unless targeted by specific interventions, they will continue to defeat any attempt of interrupting malaria transmission. These may be represented by either mass drug administration or mass screening and treatment. However, for the latter a sufficiently sensitive and field-adapted diagnostic test able to detect sub-patent infections would be needed. The choice of the approach to be taken will depend on its feasibility according to the local conditions and the percentage of infected people. Above a given malaria prevalence, mass drug administration may be more appropriate and cost-effective than mass screening and treatment.
